# Supplementary material for: Structure of mammalian Mediator complex reveals Tail module architecture and interaction with a conserved core
Source: Nat Commun. 2021 Mar 1;12:1355. doi: 10.1038/s41467-021-21601-w (PMC7921410; doi:10.1038/s41467-021-21601-w)
Supplement: Supplementary file 1 — Supplementary Information [file 41467_2021_21601_MOESM1_ESM.pdf]

Structure of mammalian Mediator complex reveals Tail module architecture and interaction with a conserved core

Haiyan Zhao[1.#], Natalie Young[1,#], Jens Kalchschmidt[2,#], Jenna Lieberman[2], Laila El Khattabi[3], Rafael Casellas[2,4] and Francisco J. Asturias[1,*]

[1]Department of Biochemistry and Molecular Genetics, University of Colorado Anschutz Medical School, Aurora CO 80045, USA

[2]Lymphocyte Nuclear Biology, NIAMS, NIH, Bethesda, MD 20892, USA

[3]Institut Cochin Laboratoire de Cytogénétique Constitutionnelle Pré et Post Natale, 75014 Paris France

[4]Center for Cancer Research, NCI, NIH, Bethesda, MD 20892, USA

[#,*]These authors contributed equally to this work

*Correspondence should be addressed to F.J.A. (Francisco.Asturias@CUAnschutz.edu))

Supplementary Table 1. MudPIT analysis of purified mMED fractions used for cryo-EM analysis

Identified Proteins	Accession Identifier	Molecular Weight	MED19-FLAG Peptide Counts
Mediator of RNA polymerase II transcription subunit 12	MED12_MOUSE	245 kDa	290
Mediator of RNA polymerase II transcription subunit 14	MED14_MOUSE	161 kDa	276
Actin, cytoplasmic 2	ACTG_MOUSE	42 kDa	241
Mediator of RNA polymerase II transcription subunit 13-like	MD13L_MOUSE	242 kDa	224
Mediator of RNA polymerase II transcription subunit 23	MED23_MOUSE	156 kDa	220
DNA-directed RNA polymerase II subunit RPB1	RPB1_MOUSE	217 kDa	216
Mediator of RNA polymerase II transcription subunit 1	MED1_MOUSE	167 kDa	163
Mediator of RNA polymerase II transcription subunit 24	MED24_MO USE	110 kDa	159
Mediator of RNA polymerase II transcription subunit 16	MED16_MOUSE	92 kDa	153
DNA-directed RNA polymerase II subunit RPB2	RPB2_MOUSE	134 kDa	136
Mediator of RNA polymerase II transcription subunit 17	MED17_MOUSE	72 kDa	132
Myosin-9	MYH9_MOUSE	226 kDa	124
Serine/threonine-protein kinase 38	STK38_MOUSE	54 kDa	117
Unconventional myosin-Ie	MYO1E_MOUSE	127 kDa	99
Actin, alpha cardiac muscle 1	ACTC_MOUSE	42 kDa	91
Mediator of RNA polymerase II transcription subunit 15	MED15_MOUSE	87 kDa	87
Gelsolin	GELS_MOUSE	86 kDa	82
Mediator of RNA polymerase II transcription subunit 27	MED27_MOUSE	35 kDa	78
Mediator of RNA polymerase II transcription subunit 4	MED4_MOUSE	30 kDa	67
Tropomodulin-3	TMOD3_MOUSE	40 kDa	67
Unconventional myosin-XVIIIa	MY18A_MOUSE	233 kDa	66
Advillin	AVIL_MOUSE	92 kDa	62
Beta-actin-like protein 2	ACTBL_MOUSE	42 kDa	62
Mediator of RNA polymerase II transcription subunit 8	MED8_MOUSE	29 kDa	57
Mediator of RNA polymerase II transcription subunit 7	MED7_MOUSE	27 kDa	53
Mediator of RNA polymerase II transcription subunit 26	MED26_MOUSE	65 kDa	52
Mediator of RNA polymerase II transcription subunit 19	MED19_MOUSE	26 kDa	52
Mediator of RNA polymerase II transcription subunit 10	MED10_MOUSE	16 kDa	48
DNA-directed RNA polymerase II subunit RPB3	RPB3_MOUSE	31 kDa	46
Mediator of RNA polymerase II transcription subunit 20	MED20_MOUSE	23 kDa	46
Mediator of RNA polymerase II transcription subunit 6	MED6_MOUSE	28 kDa	45
Cyclin-dependent kinase 8	CDK8_MOUSE	53 kDa	42
Mediator of RNA polymerase II transcription subunit 11	MED11_MOUSE	13 kDa	42
Mediator of RNA polymerase II transcription subunit 21	MED21_MOUSE	16 kDa	35
DNA-directed RNA polymerase II subunit RPB7	RPB7_MOUSE	19 kDa	35

Mediator of RNA polymerase II transcription subunit 28	MED28_MOUSE	20 kDa	34
Mediator of RNA polymerase II transcription subunit 30	MED30_MOUSE	20 kDa	34
Mediator of RNA polymerase II transcription subunit 22	MED22_MOUSE	22 kDa	33
Cyclin-dependent kinase 19	CDK19_MOUSE	57 kDa	33
Thyroid hormone receptor-associated protein 3	TR150_MOUSE	108 kDa	30
Mediator of RNA polymerase II transcription subunit 18	MED18_MOUSE	24 kDa	28
Unconventional myosin-Ic	MYO1C_MOUSE	122 kDa	28
DNA-directed RNA polymerases I, II, and III subunit RPABC1	RPAB1_MOUSE	25 kDa	27
Mediator of RNA polymerase II transcription subunit 29	MED29_MOUSE	21 kDa	27
F-actin-capping protein subunit beta	CAPZB_MOUSE	31 kDa	27
Unconventional myosin-Ig	MYO1G_MOUSE	117 kDa	27
Mediator of RNA polymerase II transcription subunit 31	MED31_MOUSE	16 kDa	25
DNA-directed RNA polymerase II subunit RPB9	RPB9_MOUSE	15 kDa	25
Mediator of RNA polymerase II transcription subunit 9	MED9_MOUSE	16 kDa	24
Actin-related protein 3	ARP3_MOUSE	47 kDa	24
Myosin regulatory light chain 12B	ML12B_MOUSE	20 kDa	23
Probable helicase senataxin	SETX_MOUSE	298 kDa	22
Myosin light polypeptide 6	MYL6_MOUSE	17 kDa	20
Calmodulin	CALM_MOUSE	17 kDa	19
Cyclin-C	CCNC_MOUSE	33 kDa	18
Heat shock cognate 71 kDa protein	HSP7C_MOUSE	71 kDa	17
DNA-directed RNA polymerase II subunit RPB4	RPB4_MOUSE	16 kDa	17
Mediator of RNA polymerase II transcription subunit 25	MED25_MOUSE	78 kDa	17
F-actin-capping protein subunit alpha-1	CAZA1_MOUSE	33 kDa	17
Keratin, type I cytoskeletal 10	K1C10_MOUSE	58 kDa	17
Actin-related protein 2	ARP2_MOUSE	45 kDa	15
DNA-directed RNA polymerases I, II, and III subunit RPABC3	RPAB3_MOUSE	17 kDa	14
Unconventional myosin-Va	MYO5A_MOUSE	216 kDa	13
Tubulin beta-5 chain	TBB5_MOUSE	50 kDa	13
Unconventional myosin-Id	MYO1D_MOUSE	116 kDa	12
DNA-directed RNA polymerase II subunit RPB11	RPB11_MOUSE	13 kDa	11
DNA-directed RNA polymerases I, II, and III subunit RPABC5	RPAB5_MOUSE	8 kDa	11
F-actin-capping protein subunit alpha-2	CAZA2_MOUSE	33 kDa	11
Actin-related protein 2/3 complex subunit 1B	ARC1B_MOUSE	41 kDa	11
Actin-related protein 2/3 complex subunit 4	ARPC4_MOUSE	20 kDa	11
Enhancer of rudimentary homolog	ERH_MOUSE	12 kDa	10
Keratin, type II cytoskeletal 1	K2C1_MOUSE	66 kDa	10
Actin-related protein 2/3 complex subunit 2	ARPC2_MOUSE	34 kDa	10

Plastin-2	PLSL_MOUSE	70 kDa	10
Keratin, type II cytoskeletal 5	K2C5_MOUSE	62 kDa	9
Keratin, type I cytoskeletal 13	K1C13_MOUSE	48 kDa	9
Keratin, type I cytoskeletal 14	K1C14_MOUSE	53 kDa	8
Keratin, type II cytoskeletal 73	K2C73_MOUSE	59 kDa	8
Bcl-2-associated transcription factor 1	BCLF1_MOUSE	106 kDa	8
Myosin light chain kinase 2, skeletal/cardiac muscle	MYLK2_MOUSE	66 kDa	7
78 kDa glucose-regulated protein	GRP78_MOUSE	72 kDa	6
Actin-related protein 2/3 complex subunit 3	ARPC3_MOUSE	21 kDa	6
Spindlin-1	SPIN1_MOUSE	30 kDa	5
Gamma-adducin	ADDG_MOUSE	79 kDa	5
Alpha-adducin	ADDA_MOUSE	81 kDa	4
Tubulin alpha-1B chain	TBA1B_MOUSE (+1)	50 kDa	4
Lymphocyte-specific protein 1	LSP1_MOUSE	37 kDa	4
E3 ubiquitin-protein ligase TRIM21	RO52_MOUSE	54 kDa	4
Elongation factor 1-alpha 1	EF1A1_MOUSE	50 kDa	3
Actin-related protein 2/3 complex subunit 5-like protein	ARP5L_MOUSE	17 kDa	3
Collagen alpha-2(I) chain	CO1A2_MOUSE	130 kDa	3
Q3TTY5|K22E_MOUSE-DECOY	Q3TTY5|K22E_MOUSE-DECOY	?	2
U4/U6 small nuclear ribonucleoprotein Prp4	PRP4_MOUSE	58 kDa	2

Mediator Subunit
RNAPII Subunit
Kinase Module Subunit
Uncommon Contaminant

Supplementary Table 2. Cryo-EM data collection, processing and model validation statistics

**Data collection and Processing**	
Microscope	Talos Arctica (ThermoFisher)
Detector	K3 Summit (Gatan)
Voltage (keV)	200
Defocus range (□m)	0.8 - 3.5
Number of movies (0°/20°/30°/40° tilt)	2,705/6,464/2,928/1,495 (13,592 total)
Frames per movie	40 - 60
Exposure time per frames (ms)	75 - 100
Magnification	36,000x
Pixel size (Å)	1.11
Dose rate (e^-/pixel/sec)	19 - 22
Total dose per movie ($e^-/Å^2$)	50 - 80
Initial number of particle images	2,319,481
Final number of particle images	217,557
Particle symmetry	C1
Overall map resolution (Å)	4.0
FSC threshold	0.143
Map resolution range (Å)	3.4 - >10
Directional resolution range (Å)	3.4 – 6.8
Sphericity of 3D FSC	0.838
Map sharpening B-factor ($Å^2$)	74
**Atomic model validation statistics**	
**Model Composition**	
Protein Chains	25
Protein Residues	7,434
Non-hydrogen atoms	47,305
**R.M.S deviations**	
Bond lengths (Å)	0.003
Bond angles (°)	0.682
**Validation**	
MolProbity score	2.07
Clashscore	8.11
Poor rotamers (%)	0
**Ramachandran plot**	
Favored (%)	86.86
Allowed (%)	13.02
Disallowed (%)	0.13

Supplementary Table 3: Subunit module assignment and atomic model information

MmMED Subunit	Chain ID	Module	Color	# of Residues	Residues in atomic model (%)
MED1	A	Middle	purple	1575	~1-520 (33)
MED4	B	Middle	light blue	270	30-129 (37)
MED6	C	Head	yellow	246	9-246 (97)
MED7	D	Middle	sandy brown	233	11-167 (67)
MED8	E	Head	forest green	268	7-186 (67)
MED9	F	Middle	hot pink	142	61-136 (53)
MED10	G	Middle	magenta	135	61-130 (52)
MED11	H	Head	orange red	117	8-115 (92)
MED14	I	-	green	1459	149-975/1172-1450 (76)
MED15	J	Tail	red	789	620-787 (21)
MED16	K	Tail	khaki	828	3-825 (99)
MED17	L	Head	dodger blue	649	15-645 (97)
MED18	M	Head	tan	208	17-203 (90)
MED19	N	Middle	navy blue	244	78-136 (24)
MED20	O	Head	cyan	212	3-200 (93)
MED21	P	Middle	dark green	144	3-128 (88)
MED22	Q	Head	medium purple	200	9-139 (66)
MED23	R	Tail	plum	1367	1-1334 (98)
MED24	S	Tail	dark cyan	987	4-985 (99)
MED25	T	Tail	salmon	745	5-216 (28)
MED26	U	Middle-Head		588	Not included
MED27	V	Tail	violet red	311	8-304 (95)
MED28	W	Tail	goldenrod	178	32-149 (66)
MED29	X	Tail	blue	199	52-185 (67)
MED30	Y	Tail	olive drab	178	27-178 (85)
MED31	Z	Middle	firebrick	131	13-105 (71)

Supplementary Table 4: Primer and cloning strategy for cell line creation.

Med14∆1206-1458 truncation in CH12 Med25Flag/Flag cells	
**sgRNA**	**sgRNA sequence**
sgRNA 1 cutting close to aa1206	TCCAAGGAATCTCTCAAGTG
sgRNA 2 cutting close to stop codon	CTATGGACGCCCACCAGGGGG

Med15-HMP overexpression in CH12 Med13-/- Med19Flag/Flag Med15-/- cells			
**PCR**	**Template DNA**	**Primer name**	**Sequence (5' - 3')**
Med15 amplification from cDNA	CH12 cDNA	Med15 mRNA F	atggacgtttcggggcagga
		Med15 mRNA R	ggcagctgagaggcaggcct
Pre-Stitch PCR1	Med15 cDNA amplification	Cterm pMY-Med15 Stitch F	GGGAATTCCTGCAGGCCGCCACCatggacgtttcggggcagga
		Cterm Med15-HMP R	CGTTAATCCAGATTACCAGTTTACCTTCTTCGATCATCCCggcagctgagaggcaggcct
Pre-Stitch PCR2	HMP-P2A-EGFP construct	Cterm Med15-HMP F	gcccagagcatccaccaggcctgcctctcagctgccGGGATGATCGAAGAAGGTAAACTG
		pMY Rev SalI	CCCGGTCGACgcggccGCTTAC
Stitch PCR	Pre-Stitch PCR1&2 products	Cterm pMY-Med15 Stitch F	GGGAATTCCTGCAGGCCGCCACCatggacgtttcggggcagga
		pMY Rev SalI	CCCGGTCGACgcggccGCTTAC

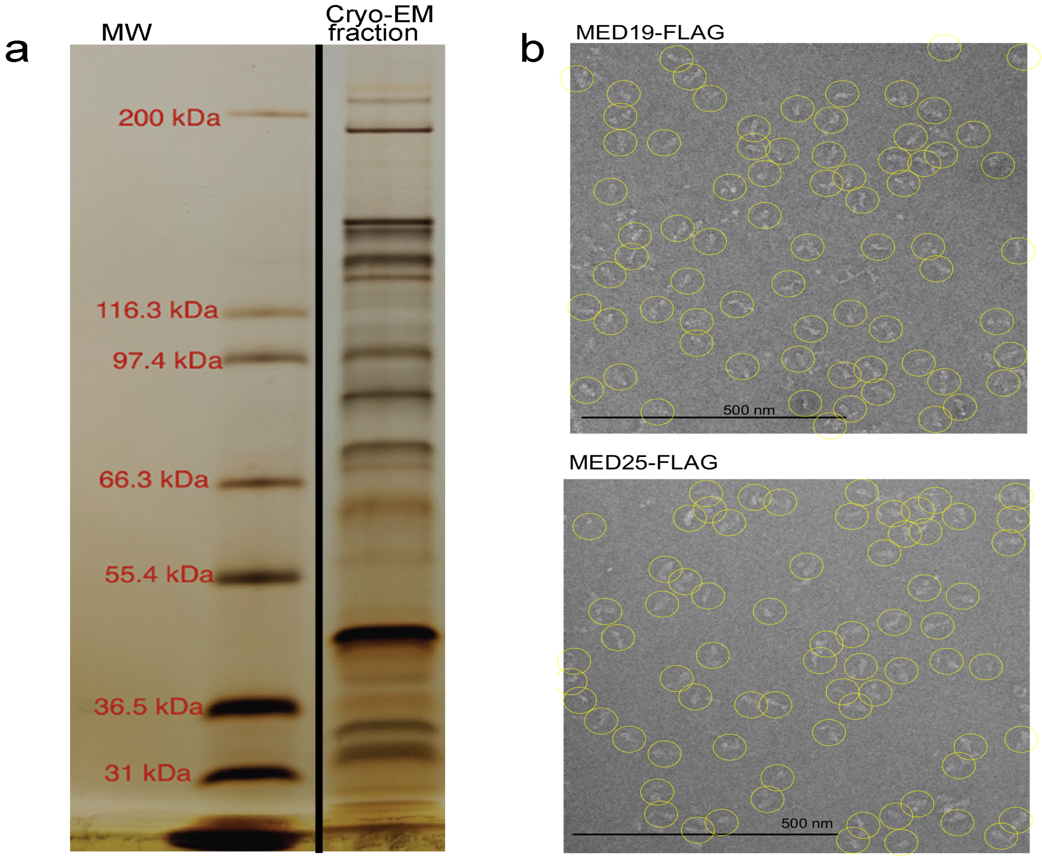

Supplementary Figure 1. SDS-PAGE and EM in stain analyses of mMED preparations used for cryo-EM. a, SDS-PAGE analysis of a typical MED25-FLAG purified mMED fraction used for cryo-EM analysis. **b,** Images of MED19-FLAG and MED25-FLAG mMED preserved in stain show homogenous particles and absence of any non-Mediator contaminants. Yellow circles highlight individual particles. Scale bars correspond to 500nm. These micrographs are representative of hundreds of micrographs used to ascertain the purity of mMED preparations used for various EM studies in stain and cryo.

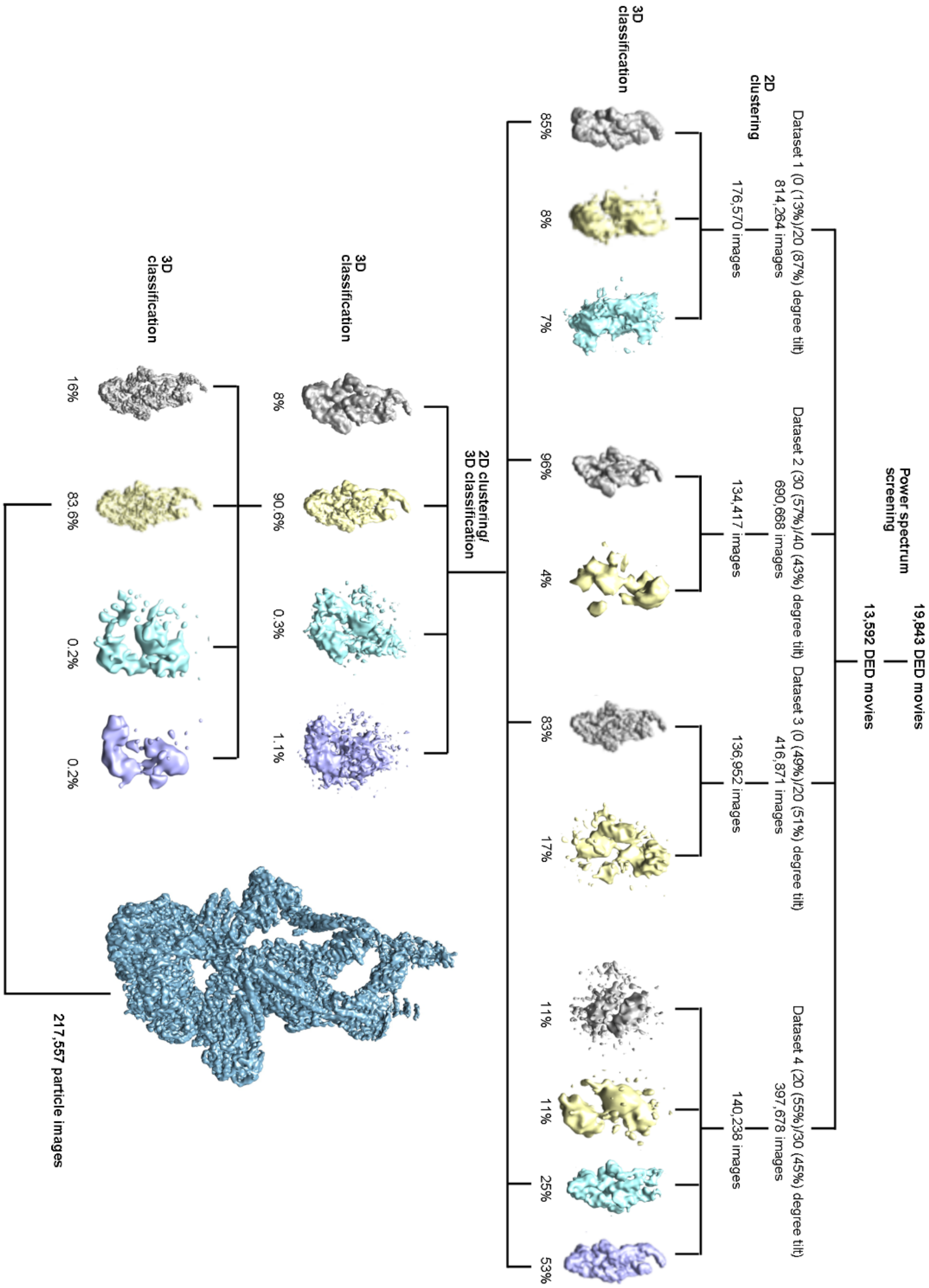

Supplementary Figure 2. 2D clustering and 3D classification diagram for mMED cryo-EM analysis. Sequence of power spectra screening, 2D clustering and 3D classification steps during selection and analysis of mMED cryo-EM particle images.

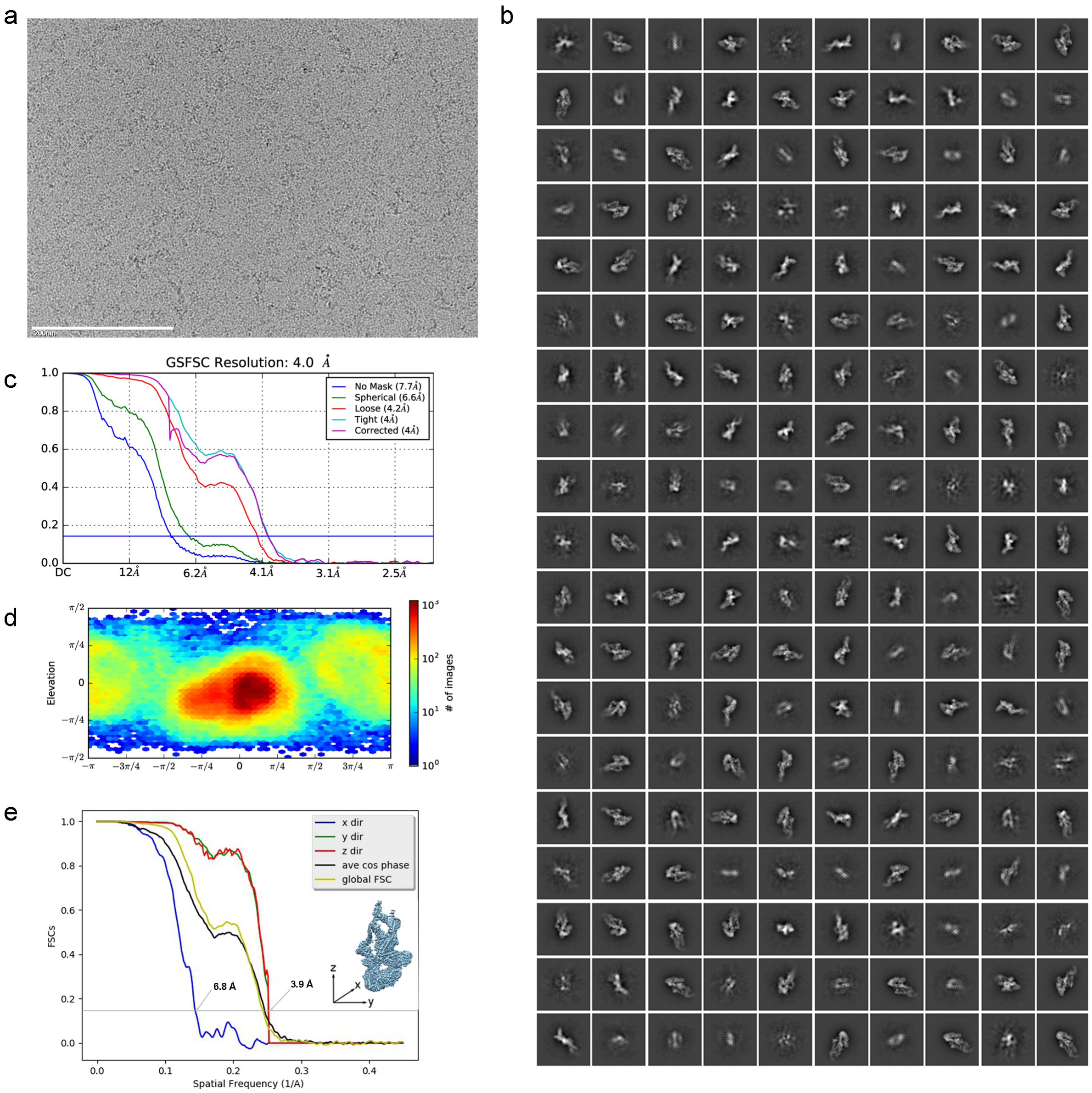

Supplementary Figure 3. mMED cryo-EM analysis. a, Typical mMED micrograph showing particles cryo-preserved on a thin carbon film (0° tilt). Scale bar 200nm. This micrograph is representative of many thousands of micrographs collected in the course of the cryo-EM studies described here. **b,** 2D class averages obtained from clustering of a combination of untilted and tilted mMED images. **c,** Fourier Shell Correlation (FSC) plot used to estimate the resolution of the mMED cryo-EM map to 4.0Å. **d,** Angular distribution plot for the final combined mMED cryo-EM dataset. **e,** Directional FSC plots showing the resolution of the mMED cryo-EM map along 3 perpendicular directions.

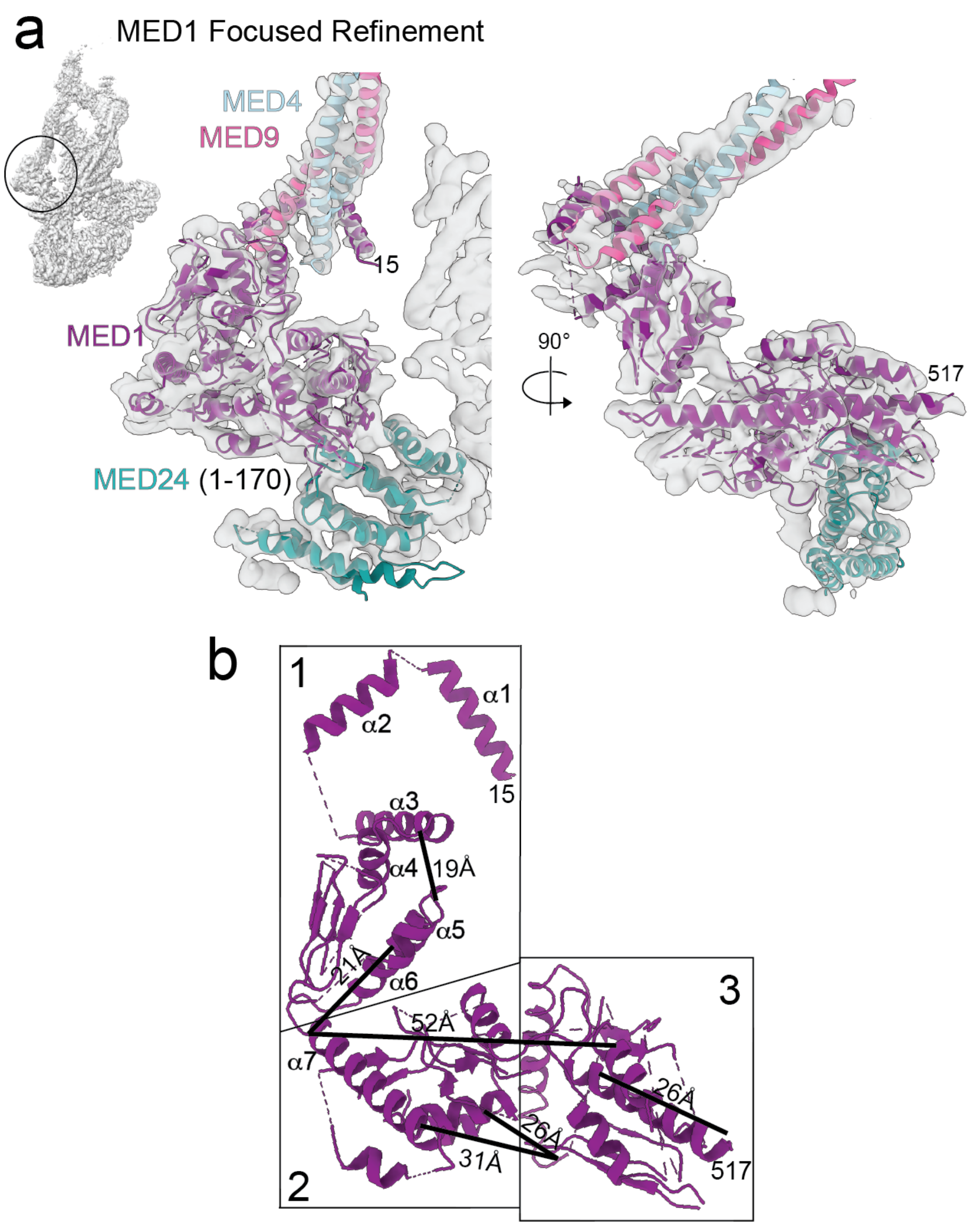

Supplementary Figure 4. Focused refinement map and structure of the MED1 portion of the Middle module. Focused refinement map including MED1 and neighboring portions of MED4, MED9, MED14 and MED24 (as indicated in left insert) and partial model of MED1 (~aa 1-520) showing its interaction with MED4-MED9 and MED24. The MED1 N-terminus wraps around the bottom portion of MED4-MED9 (in light blue and pink, respectively) and a N-terminal α-helix contacts both subunits. From there, alternating β-strand and α-helical domains extend to contact the very N-terminal portion of MED24 (in dark cyan) through an extended β-sheet domain. With its alternating arrangement of helices and strands, the structure of the ordered N-terminal portion of MED1 is reminiscent of MED14 and could undergo conformational rearrangements allowing the Middle to move without breaking its contact with MED24.

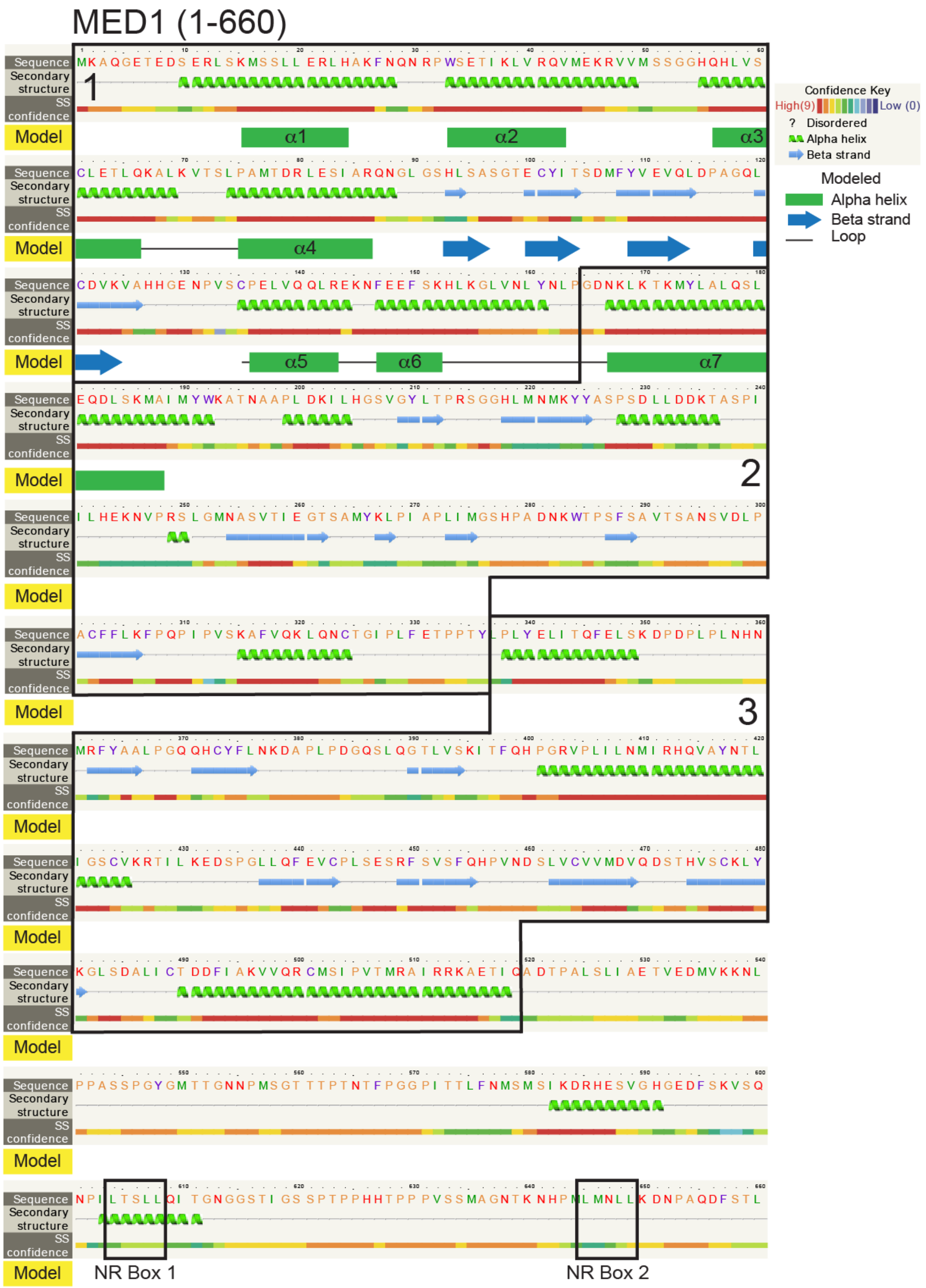

Supplementary Figure 5. Secondary structure prediction for the N-terminal portion of MED1 in the Middle module. Secondary structure prediction for the MED1 N-terminal region (aa 1-660) calculated using Phyre2[35]. Three regions, delineated as indicated by black outlines, correspond to portions of the partial MED1 model shown in Supplementary Fig. 4. Region 3 includes β-strands that interact directly with a hydrophobic patch on the MED24 N-terminus. No ordered domains are predicted after the first ~700 aa, which means that the entire C-terminal portion of MED1 (~900 residues or >55% of the 1575 aa protein) are disordered.

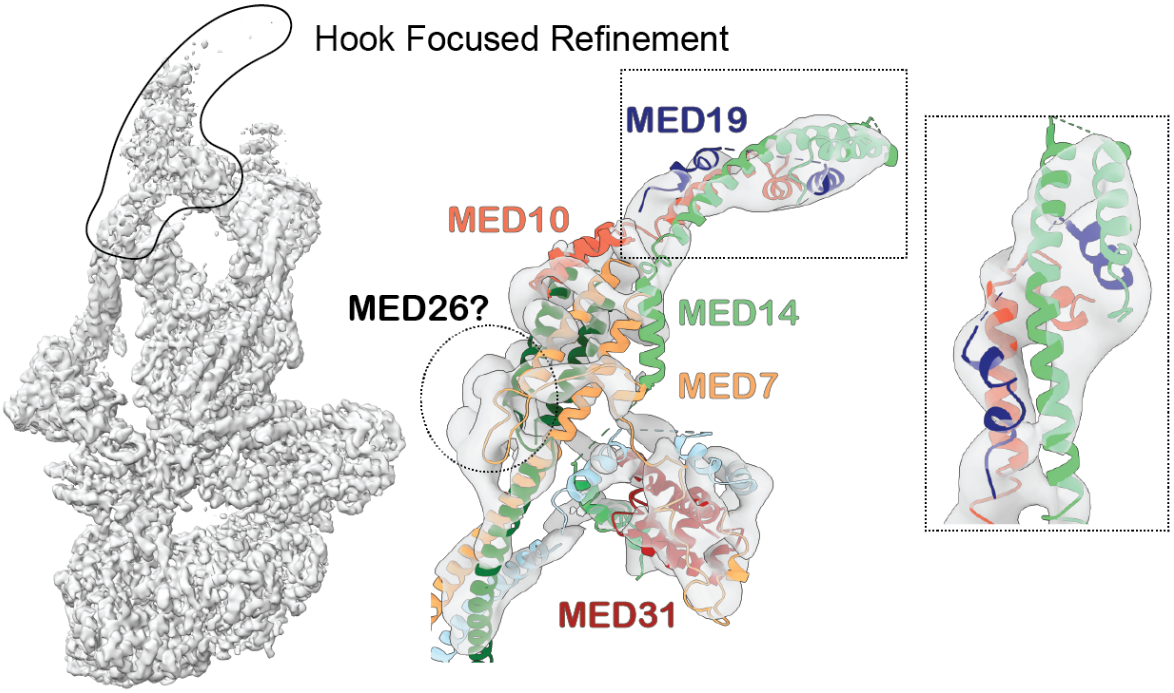

Supplementary Figure 6. Focused refinement map and structure of the hook portion of the Middle module. Focused refinement map of the hook/knob portion of the Middle module and models of the corresponding subunits fitted into the map.

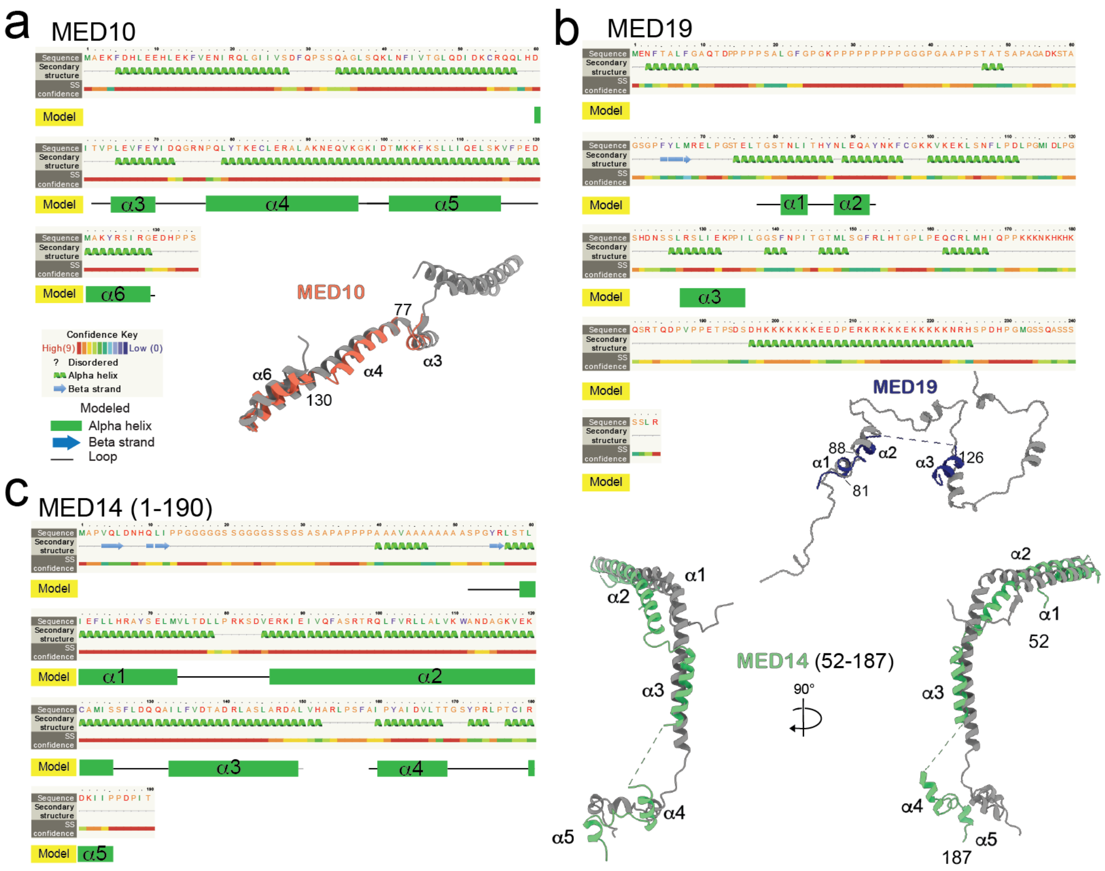

Supplementary Figure 7. Secondary structure prediction for the MED1 portion of the Middle module. Secondary structure predictions (from Phyre2[35]) for MED10, MED19 and MED14's 150 N-terminal residues show them to be mostly helical and to correspond both in size and expected secondary structure with corresponding *S pombe* Mediator subunits (MED19 is larger in mouse than yeast). Molecular models of mammalian hook subunits (MED14 N-terminus, MED10, MED19) based on secondary structure predictions and the focused refinement map show structures that match well corresponding portions of the atomic model of *S pombe* Mediator (PDB 5N9J).

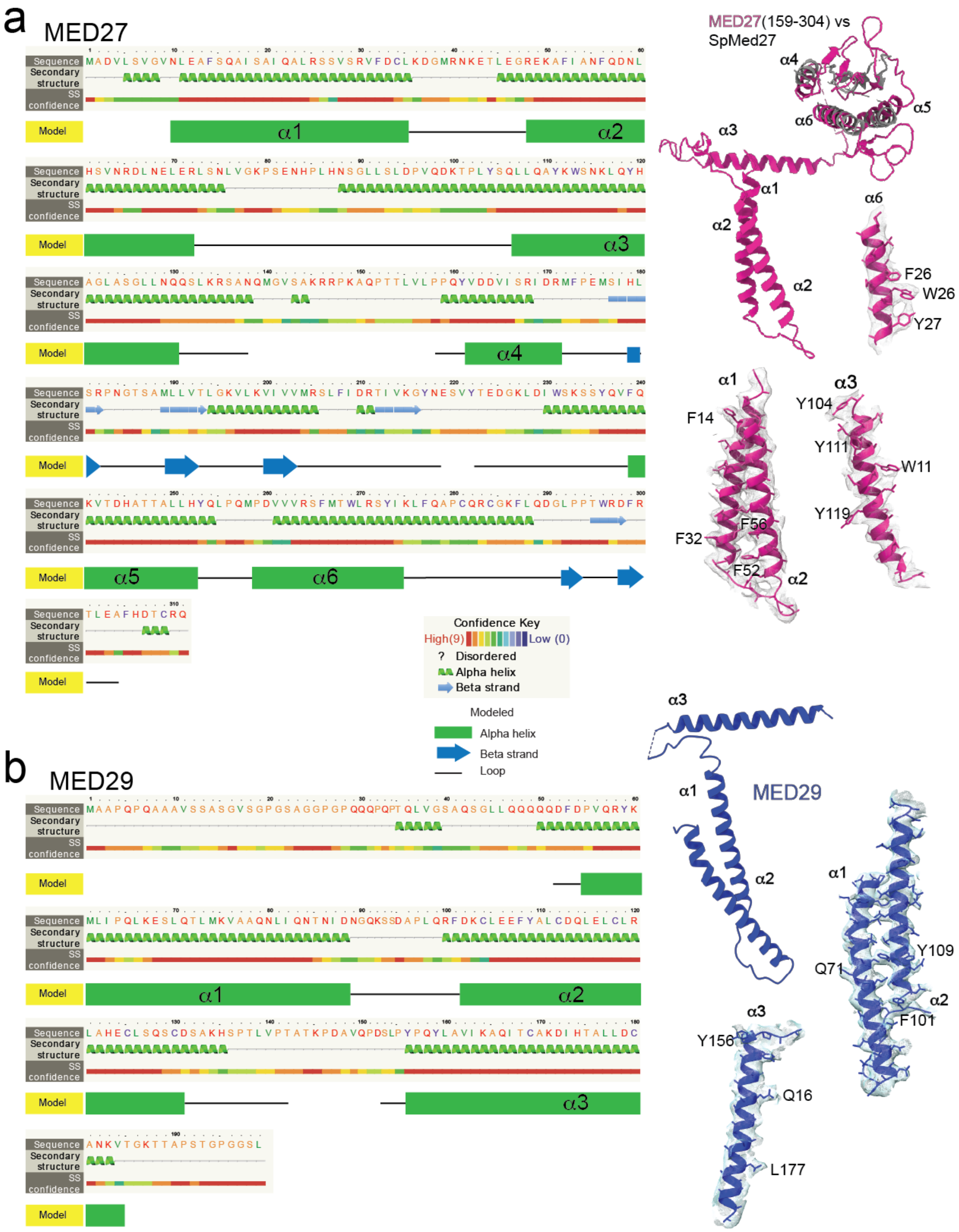

Supplementary Figure 8. Interpretation and structure of upper Tail subunits MED27/MED29. Secondary structure predictions for MED27/MED29 (left) calculated using Phyre2[35] show that both subunits (which are very similar in size) are expected to have the same overall secondary structure, but that the length of specific α-helices and loops differs between them. MED27 also has a unique globular C-terminal domain. This, and clear bulky side-chain densities in the corresponding portions of the mMED cryo-EM map (right) made possible building an accurate model of these two upper Tail subunits.

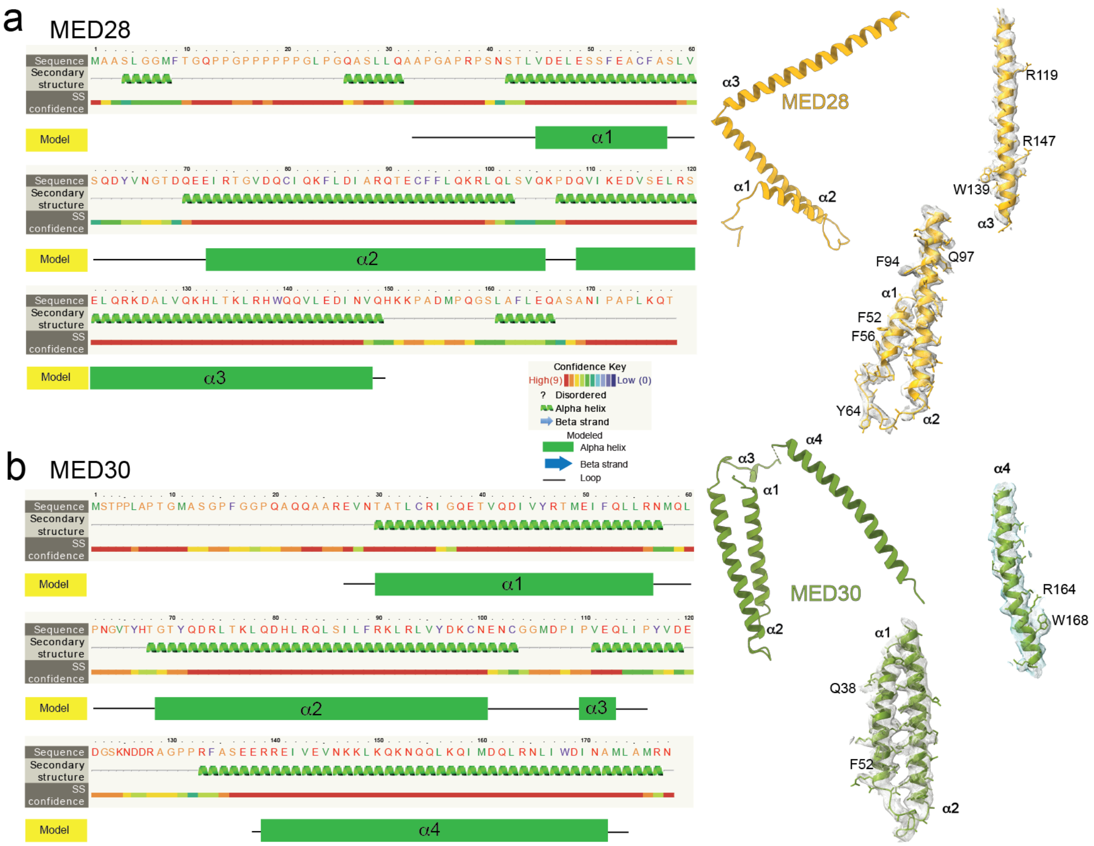

Supplementary Figure 9. Interpretation and structure of upper Tail subunits MED28/MED30. Secondary structure predictions for MED28/MED30 (left) calculated using Phyre2[35] show that both subunits (which are very similar in size) are expected to have the same overall secondary structure, but that the length of specific α-helices and loops differs between them. This, and clear bulky side-chain densities in the corresponding portions of the mMED cryo-EM map (right) made possible building an accurate model of these two upper Tail subunits.

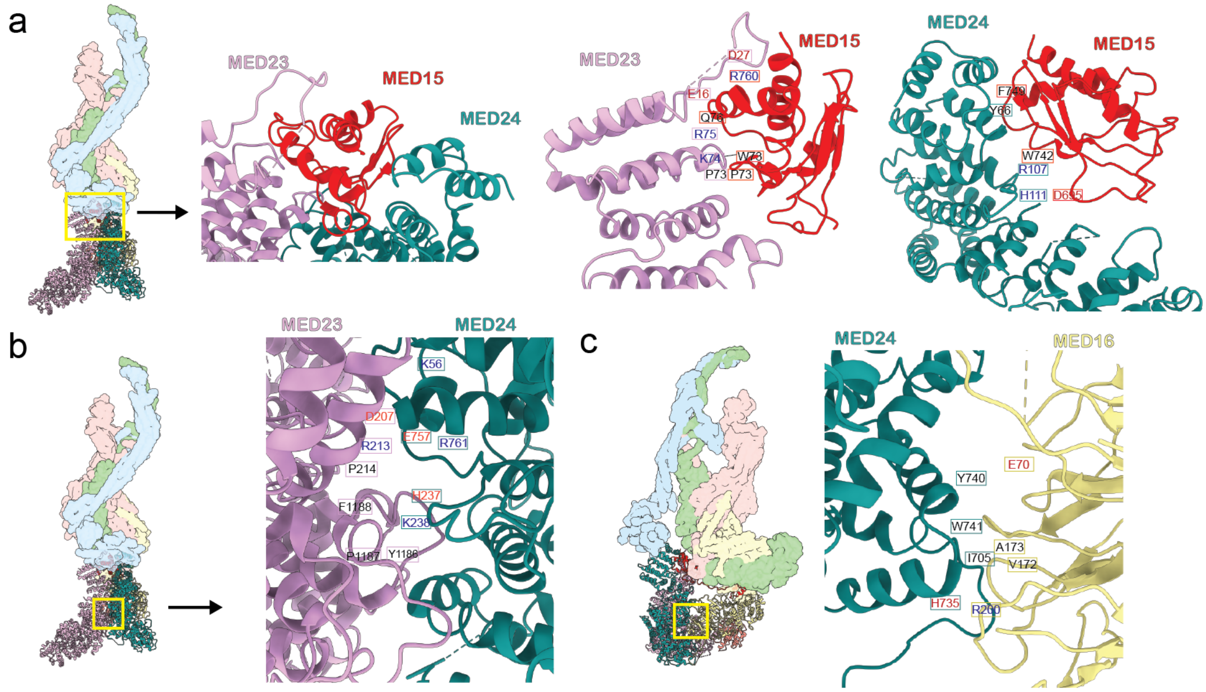

Supplementary Figure 10. Inter-subunit contacts in the lower Tail. Lower Tail subunit interfaces show a number of charged, hydrophobic and cationic-π interactions, which are depicted by coloring the involved residues in black (polar and hydrophobic amino acids), red (negatively charged) and blue (positively charged). **a,** Interaction of MED15 with MED23 and MED24. The MED15/MED23 interface shows electrostatic interactions between MED23 D27, E16, R75 and MED15 R760 and Q763, with potential hydrogen-bonding between MED23 E16 and MED15 Q763. There is a cationic-π interaction between MED23 K74 and MED15 W737, followed by hydrophobic-interaction between MED23 P73 and MED15 P735. The MED15/MED24 interface includes hydrophobic (MED24 Y66-MED15 F749), cationic-π (MED24 R107-MED15 W742), and electrostatic (MED24 H111-MED15 D695) interactions. **b,** In the MED23/MED24 interface. MED23 D207 interacts with K56 and R761 on MED24 to form an electrostatic interface, with a potential salt-bridge between MED23 R213 and MED24 E757. Below this interaction, there is an aromatic cluster formed by MED23 P214, F1188, P1187, Y1186 and MED24 H237, with a cationic-π interaction between MED23 F1188 and MED24 K238. **c,** At the MED16/MED24 interface, MED24 Y740 forms a hydrogen-bond with E70 of MED16. A hydrophobic patch between MED24 W741, I705 and MED16 A173, V172 is observed, as well as a charge interaction between MED24 H735 and MED16 R200.

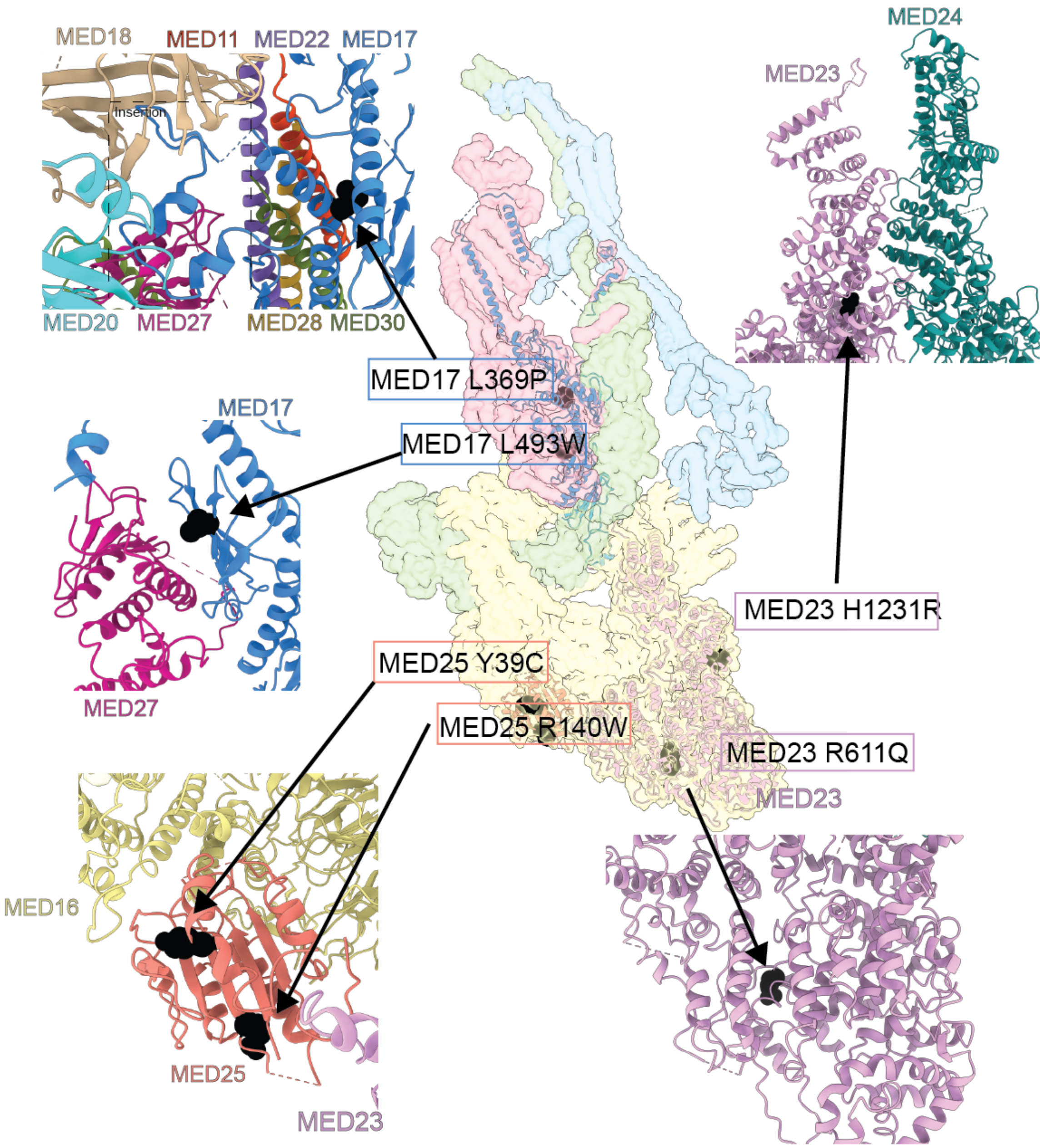

Supplementary Figure 11. Disease-associated mMED mutations and mMED subunit IDRs. a, Location of known disease-associated mammalian Mediator mutations.
