# Supplementary material for: Structure of mammalian Mediator complex reveals Tail module architecture and interaction with a conserved core
Source: Nat Commun. 2021 Mar 1;12:1355. doi: 10.1038/s41467-021-21601-w (PMC7921410; doi:10.1038/s41467-021-21601-w)
Supplement: Supplementary file 4 — Reporting Summary [file 41467_2021_21601_MOESM4_ESM.pdf]

nature research

Corresponding author(s): Francisco J Asturias

Last updated by author(s): 01/22/2021

Reporting Summary

Nature Research wishes to improve the reproducibility of the work that we publish. This form provides structure for consistency and transparency in reporting. For further information on Nature Research policies, see Authors & Referees and the Editorial Policy Checklist.

Statistics

For all statistical analyses, confirm that the following items are present in the figure legend, table legend, main text, or Methods section.

n/a	Confirmed	
☐	☒	The exact sample size (*n*) for each experimental group/condition, given as a discrete number and unit of measurement
☐	☒	A statement on whether measurements were taken from distinct samples or whether the same sample was measured repeatedly
☒	☐	The statistical test(s) used AND whether they are one- or two-sided<br>*Only common tests should be described solely by name; describe more complex techniques in the Methods section.*
☒	☐	A description of all covariates tested
☐	☒	A description of any assumptions or corrections, such as tests of normality and adjustment for multiple comparisons
☐	☒	A full description of the statistical parameters including central tendency (e.g. means) or other basic estimates (e.g. regression coefficient) AND variation (e.g. standard deviation) or associated estimates of uncertainty (e.g. confidence intervals)
☒	☐	For null hypothesis testing, the test statistic (e.g. *F*, *t*, *r*) with confidence intervals, effect sizes, degrees of freedom and *P* value noted<br>*Give P values as exact values whenever suitable.*
☒	☐	For Bayesian analysis, information on the choice of priors and Markov chain Monte Carlo settings
☒	☐	For hierarchical and complex designs, identification of the appropriate level for tests and full reporting of outcomes
☒	☐	Estimates of effect sizes (e.g. Cohen's *d*, Pearson's *r*), indicating how they were calculated

Our web collection on statistics for biologists contains articles on many of the points above.

Software and code

Policy information about availability of computer code

Data collection	Automated cryo-EM data collection was carried out using the Leginon/Appion software package
Data analysis	Cryo-EM data analysis was done using the CryoSPARC v2.8.3, RELION 2.1, Matlab 9.7 and EMAN2.22/SPARX data analysis packages. Secondary and tertiary structure predictions were done using Phyre2 V 2.2 and I-TASSER. Cryo-EM map interpretation and model building/refinement were done using the Chimera 1.13.1 and ChimeraX 0.94, Coot 0.8.9.2 and Phenix 1.16 software packages

For manuscripts utilizing custom algorithms or software that are central to the research but not yet described in published literature, software must be made available to editors/reviewers. We strongly encourage code deposition in a community repository (e.g. GitHub). See the Nature Research guidelines for submitting code & software for further information.

Data

Policy information about availability of data

All manuscripts must include a data availability statement. This statement should provide the following information, where applicable:

- Accession codes, unique identifiers, or web links for publicly available datasets
- A list of figures that have associated raw data
- A description of any restrictions on data availability

Data availability
Cryo-EM maps and atomic coordinates have been deposited with the Electron Microscopy Data Bank (with accession code EMD-21514 and Protein Data Dank (accession code PDB ID 6W1S).

Field-specific reporting

Please select the one below that is the best fit for your research. If you are not sure, read the appropriate sections before making your selection.

☒ Life sciences ☐ Behavioural & social sciences ☐ Ecological, evolutionary & environmental sciences

For a reference copy of the document with all sections, see nature.com/documents/nr-reporting-summary-flat.pdf

Life sciences study design

All studies must disclose on these points even when the disclosure is negative.

Sample size	For cryo-EM image data, the number of images was determined based on resolution and angular distribution criteria. The number of images included in the analysis was selected to maximize resolution under the chosen experimental conditions and to ensure an even distribution of views as required to calculate a 3D map free of reconstruction artifacts.
Data exclusions	No cryo-EM data was excluded from the analysis, except for application of standard protocols (2D image clustering) for elimination of non-particle images included in the initial set of images obtained by automated particle selection. The final subset of cryo-EM images used for calculation of the mammalian Mediator 3D map was further refined using standard 3D classification protocols to maximize the resolution of the cryo-EM map.
Replication	As is standard in cryo-EM analysis, our image dataset was split only to obtain an objective measurement of map resolution. Validation of the final map was aided by comparison with known structures of yeast Mediator complexes, consideration of consistency with known standard secondary structure elements, and correspondence to features expected based on primary-sequence-based structure predictions.
Randomization	This is not applicable to the analysis involved in determination of the mammalian Mediator cryo-EM structure, which relies on computational analysis of many thousands of cryo-EM images of individual Mediator particles.
Blinding	This is not applicable to the analysis involved in determination of the mammalian Mediator cryo-EM structure, as we were not seeking to establish changes in structure triggered by a specific factor, but just determine the structure of the Mediator complex.

Reporting for specific materials, systems and methods

We require information from authors about some types of materials, experimental systems and methods used in many studies. Here, indicate whether each material, system or method listed is relevant to your study. If you are not sure if a list item applies to your research, read the appropriate section before selecting a response.

Materials & experimental systems

n/a	Involved in the study	
☒	☐	Antibodies
☐	☒	Eukaryotic cell lines
☒	☐	Palaeontology
☒	☐	Animals and other organisms
☒	☐	Human research participants
☒	☐	Clinical data

Methods

n/a	Involved in the study	
☒	☐	ChIP-seq
☒	☐	Flow cytometry
☒	☐	MRI-based neuroimaging

Eukaryotic cell lines

Policy information about cell lines

Cell line source(s)	CH12 B cell lymphoma mouse cell line from Prof Tasuku Honjo, Kyoto University Japan
Authentication	All engineered cell lines were single cell subcloned and genotyped.
Mycoplasma contamination	none
Commonly misidentified lines (See ICLAC register)	No commonly misidentified cell lines were used in this study
