# Supplementary material for: Structure of mammalian Mediator complex reveals Tail module architecture and interaction with a conserved core
Source: Nat Commun. 2021 Mar 1;12:1355. doi: 10.1038/s41467-021-21601-w (PMC7921410; doi:10.1038/s41467-021-21601-w)
Supplement: Supplementary file 5 — Description of Additional Supplementary Files [file 41467_2021_21601_MOESM5_ESM.docx]

Description of additional supplementary files

Title: Supplementary Movie 1

Description: A movie showing the mMED atomic model, including a close up view of the Tail module.
